# Radiomic Machine-Learning Classifiers for Prognostic Biomarkers of Head and Neck Cancer

**DOI:** 10.3389/fonc.2015.00272

**Published:** 2015-12-03

**Authors:** Chintan Parmar, Patrick Grossmann, Derek Rietveld, Michelle M. Rietbergen, Philippe Lambin, Hugo J. W. L. Aerts

**Affiliations:** ^1^Department of Radiation Oncology, Dana-Farber Cancer Institute, Brigham and Women’s Hospital, Harvard Medical School, Boston, MA, USA; ^2^Department of Radiology, Dana-Farber Cancer Institute, Brigham and Women’s Hospital, Harvard Medical School, Boston, MA, USA; ^3^Radiation Oncology (MAASTRO), Research Institute GROW, Maastricht University, Maastricht, Netherlands; ^4^Department of Biostatistics and Computational Biology, Dana-Farber Cancer Institute, Boston, MA, USA; ^5^Department of Radiation Oncology, VU University Medical Center, Amsterdam, Netherlands; ^6^Department of Otolaryngology/Head and Neck Surgery, VU University Medical Center, Amsterdam, Netherlands

**Keywords:** quantitative imaging, radiology, radiomics, cancer, machine learning, computational science

## Abstract

**Introduction:**

“Radiomics” extracts and mines a large number of medical imaging features in a non-invasive and cost-effective way. The underlying assumption of radiomics is that these imaging features quantify phenotypic characteristics of an entire tumor. In order to enhance applicability of radiomics in clinical oncology, highly accurate and reliable machine-learning approaches are required. In this radiomic study, 13 feature selection methods and 11 machine-learning classification methods were evaluated in terms of their performance and stability for predicting overall survival in head and neck cancer patients.

**Methods:**

Two independent head and neck cancer cohorts were investigated. Training cohort HN1 consisted of 101 head and neck cancer patients. Cohort HN2 (*n* = 95) was used for validation. A total of 440 radiomic features were extracted from the segmented tumor regions in CT images. Feature selection and classification methods were compared using an unbiased evaluation framework.

**Results:**

We observed that the three feature selection methods minimum redundancy maximum relevance (AUC = 0.69, Stability = 0.66), mutual information feature selection (AUC = 0.66, Stability = 0.69), and conditional infomax feature extraction (AUC = 0.68, Stability = 0.7) had high prognostic performance and stability. The three classifiers BY (AUC = 0.67, RSD = 11.28), RF (AUC = 0.61, RSD = 7.36), and NN (AUC = 0.62, RSD = 10.52) also showed high prognostic performance and stability. Analysis investigating performance variability indicated that the choice of classification method is the major factor driving the performance variation (29.02% of total variance).

**Conclusion:**

Our study identified prognostic and reliable machine-learning methods for the prediction of overall survival of head and neck cancer patients. Identification of optimal machine-learning methods for radiomics-based prognostic analyses could broaden the scope of radiomics in precision oncology and cancer care.

## Introduction

The emergence of “radiomics” (1) has expanded the scope of medical imaging in clinical oncology. Radiomics focuses on extracting and mining a large number of medical imaging features. It is hypothesized that these imaging features are enriched with crucial information regarding tumor phenotype (1, 2). These features provide a comprehensive characterization of entire tumors, and hence are likely to capture the intra-tumor heterogeneity. It has been stated that intra-tumor heterogeneity could have profound implications in clinical predictions (e.g., treatment response, survival outcomes, disease progression, etc.), and therefore it is considered as a crucial factor for precision oncology and related research (3–6). Several studies have assessed various radiomic features in different cancer types and with respect to different imaging modalities (2, 7–11). Some studies have investigated the reproducibility/variability of radiomic features across different clinical settings (2, 12–14). Moreover, several other studies have reported significant predictive/prognostic power of radiomic features. It has been shown that radiomic features are associated with tumor histology (15–17), tumor grades or stages (16), patient survival (2, 7, 18–20), metabolism (21), and various other clinical outcomes (7, 16, 22, 23). Furthermore, some radio-genomic studies have reported associations between radiomic features and underlying gene expression patterns (2, 9, 11, 24, 25). These reports indicate that radiomics could improve individualized treatment selection and monitoring. Furthermore, unlike most of the genomic-based approaches, radiomics is non-invasive and relatively cost-effective (2, 26). Therefore, radiomics is a novel and promising step forward toward the realization of precision oncology.

Predictive and prognostic models are an important part of radiomics (27). Highly accurate and reliable models are desired to improve decision support in clinical oncology. Machine learning could help in this regard. Machine learning can be broadly defined as computational methods/models using data to improve performance or make accurate predictions (28). These programmable methods can “learn” from the data, and hence automate and improve the prediction process. Therefore, it is essential to compare different machine-learning models for precision oncology, and hence also for radiomics-based clinical biomarkers. Recent advances in medical image acquisition technologies allow higher resolution tumor imaging and facilitate detailed quantification of tumor phenotype. The feature dimensions of radiomics are increasing rapidly. One of the issues with high dimensional feature space is the “curse of dimensionality” (29). A large number of features with limited sample size could hinder the predictive/prognostic power of a model. Feature/variable selection is one of the ways to tackle the curse of dimensionality. Therefore, different feature selection methods (29) should be thoroughly investigated for radiomics-based prognostic analyses. However, as radiomics is an emerging field of research, not sufficient effort could be made toward assessing the impact of different machine-learning methods. The majority of the radiomics-based studies have only assessed the discriminating power of radiomic features without evaluating alternative prediction/prognostic models.

Only few recent studies have compared different feature selection and classification methods on radiomics-based clinical predictions (15, 20), but with limited sample sizes and also without independent validation. In a recently published radiomic study (30), a large panel of feature selection and machine-learning classification methods was evaluated in two independent cohorts of patients with non-small cell lung cancer (NSCLC). They proposed an unbiased framework for comparing different feature selection and classification methods using publicly available implementations (31, 32) and reported parameter configurations (33).

In this study, we assessed a large panel of machine-learning methods for overall survival prediction of head and neck cancer (HNSCC) patients. Two independent HNSCC cohorts totaling 196 patients were used in the analysis. Feature selection and classification training was done using training cohort HN1 and the prediction performance was evaluated in the validation cohort HN2. All the feature selection and classification methods were evaluated in terms of their prognostic ability and stability against data perturbation. Machine-learning methods having high prognostic/predictive power and stability are desired for radiomics-based analyses. Such methods could enhance the applications of non-invasive and cost-effective radiomics in cancer care.

## Materials and Methods

### Radiomic Features

We used 440 radiomic features describing the first order intensity statistics, texture (34, 35), and shape of the three-dimensional tumor region on CT images. Intensity and textural features were also recomputed after different wavelet decomposition of the original image. Mathematical definitions of all radiomic features as well as the extraction methods were previously described (2, 30).

### Datasets

In this study, we analyzed two HNSCC cohorts from the two different institutes of Netherlands:

(1) HN1: 136 HNSCC patients treated at MAASTRO Clinic, Maastricht. All patients received a treatment planning ^18F^FDG-PET-CT scan (Biograph, SOMATOM Sensation-16 with an ECAT ACCEL PET scanner; Siemens, Erlangen, Germany) made with the patient immobilized using a thermoplastic mask. Patients fasted at least 6 hours before the start of the acquisition. A total dose dependent on the weight of the patient (weight ×4 + 20 MBq) of [18F] fluoro-2-deoxy-d-glucose (FDG) 30 (MDS Nordion, Liège, Belgium), was injected intravenously, followed by physiological saline (10 mL). Free-breathing PET and CT images were acquired after an uptake period of 45 min. A spiral CT (3 mm slice thickness) was performed covering the complete thoracic region. Based on the radiological examinations and clinical findings, the gross tumor volume (GTV) was delineated on the fused PET-CT scan by a radiation oncologist in a radiotherapy treatment planning system (XiO, CMS, St Louis, MO, USA).
(2) HN2: 95 HNSCC patients treated at VU University Medical Center (VUMC), Amsterdam. All patients received a treatment planning CT scan of the head and neck (Varian Medical Systems VISION 3253). CT scans were acquired in helical mode with slice thickness of 2.5 mm. The GTV was delineated by an experienced radiation oncologist on the CT scans.

This analysis was carried out in accordance with Dutch law. The Institutional Review Boards of both the participating centers approved the studies: HN1 (MAASTRO Clinic Maastricht, The Netherlands) and HN2 (VUMC, Amsterdam, The Netherlands). Further details related to patient population and treatments can be obtained from the previous study (2). We dichotomized the censored continuous survival data using a cutoff time of 3 years. Patients who survived beyond the cutoff time were labeled as 1, whereas the deceased ones were labeled as 0. The objective of the study was to stratify patients into these two labeled survival classes using different machine-learning classifiers. We used 3 years survival cut-off because it resulted in reasonable event ratios (37% for HN1, 34% for HN2) in the cohorts. We excluded patients that were followed up for <3 years. This resulted in 101 patients in the training cohort (HN1) and 95 patients in the validation cohort (HN2). All features were standardized using *Z*-score standardization.

### Feature Selection Methods

As described in a previously published study (30), 14 FS methods based on filter approaches were used in the analysis: Fisher score (FSCR), relief (RELF), *T*-score (TSCR), Chi-square (CHSQ), Wilcoxon (WLCX), Gini index (GINI), mutual information maximization (MIM), mutual information feature selection (MIFS), minimum redundancy maximum relevance (MRMR), conditional infomax feature extraction (CIFE), joint mutual information (JMI), conditional mutual information maximization (CMIM), interaction capping (ICAP), and double input symmetric relevance (DISR). However, the method CHSQ did not run according to our experimental design. CHSQ was not able to select the required number of features due to the smaller size of training cohort. We thus removed it from further analysis. The acronyms related to the feature selection methods are defined in Table 1. Publicly available implementations were used for these methods (31, 32). Detailed description regarding these methods can be obtained from Parmar et al. (30).

**Table 1:** **Table defining the acronyms related to the used feature selection and classification methods**.

Classification method acronym	Classification method name	Feature Selection method acronym	Feature selection method name
Nnet	Neural network	RELF	Relief
DT	Decision tree	FSCR	Fisher score
BST	Boosting	GINI	Gini index
BY	Bayesian	JMI	Joint mutual information
BAG	Bagging	CIFE	Conditional infomax feature extraction
RF	Random forset	DISR	Double input symmetric relevance
MARS	Multi adaptive regression splines	MIM	Mutual information maximization
SVM	Support vector machines	CMIM	Conditional mutual information maximization
NN	Neirest neighbor	ICAP	Interaction capping
GLM	Generalized linear models	TSCR	*T*-test score
PLSR	Partial least squares and prinicipal component regression	MRMR	Minimum redundancy maximum relevance
–	–	MIFS	Mutual information feature selection
–	–	WLCX	Wilcoxon

### Classifiers

As described earlier (30), we investigated 12 machine-learning classifiers belonging to the 12 classifier families: bagging (BAG), Bayesian (BY), boosting (BST), decision trees (DT), discriminant analysis (DA), generalized linear models (GLM), multiple adaptive regression splines (MARS), nearest neighbors (NN), neural networks (Nnet), partial least square and principle component regression (PLSR), random forests (RF), and support vector machines (SVM). In our experimental settings, classifier DA generated computation error in the majority of cases. This could be due to the smaller training cohort. Therefore, we removed DA from further analysis and used the remaining 11 classifiers. The acronyms related to the classifiers are defined in Table 1. All classifiers were implemented using the R package caret (version 6.0-47) (36), which provides a nice interface to access many machine-learning algorithms in R. Classifiers were trained using the repeated (three repeat iterations) 10-fold cross validation in the training cohort (HN1), and their prognostic performance was evaluated in the validation cohort (HN2) using the area under receiver operator characteristic (ROC) curve (AUC). We used the classifier parameters as defined by earlier studies (30, 33). All the classifiers, the corresponding parameters and R packages are listed in Ref. (30).

### Analysis

#### Prognostic Performance

We compared different feature selection and classification methods using the experimental design defined by an earlier study of NSCLC radiomic cohorts (30). We incrementally selected features ranging from 5 up to 50, with an increment of 5 features (*n* = 5, 10, 15, 20, … , 50), using each of the 13 feature selection methods. These subsets of selected features were then used as an input to each of the 11 machine-learning classifiers. The prognostic performance was assessed using the area under receiver operator characteristic curve (AUC).

#### Stability

Stability of feature selection and classification methods was assessed using previously defined stability measures (stability and RSD) (30). Stability measures were computed using the training cohort (HN1), and results were reported as median ± SD of 100 bootstrap iterations. It should be noted that in order to compute classifier stability, we first selected 30 representative features using MRMR. These selected features were then used as classifier input while computing classifier stability (RSD). We used MRMR because it showed the highest prognostic performance among all feature selection methods.

#### Prognostic Performance and Stability

As similar to Ref. (30), we used the median values of AUC and stability as thresholds to categorize the feature selection and classification methods into low or high performance (stability) groups. We created two rank lists based on AUC and stability and cited the methods as highly accurate and reliable, which ranked in the top half (greater than or equal to median value) in both ranked lists. Feature selection methods with stability ≥0.66 (median stability of all feature selection methods) and AUC ≥ 0.61 (median AUC of all feature selection methods) are considered as highly reliable and accurate methods. Similarly, classification methods with RSD ≤ 11.4 (median RSD of all classifiers) and AUC ≥ 0.61 (median AUC of all classifiers) are considered as highly reliable and accurate ones.

#### Experimental Factors Affecting Radiomics-Based Survival Prediction

Multifactor analysis of variance (ANOVA) was used to assess the variability in survival prediction. Three experimental factors were considered for the variability analysis: feature selection method, classification method, and the number of selected features. In order to compare the variability contributed by each factor and their interactions, the estimated variance components were divided by the total variance.

### Comparison with the NSCLC Cohort Study

The results of this study were relatively compared with the previously published study of NSCLC radiomic cohorts (30). For both NSCLC and HNSCC studies, all methods were categorized into two groups: low (less than threshold) or high (greater than threshold). This grouping was carried out using the corresponding threshold values (median AUC and median stability). A method was considered consistent, if it belonged to the same group (high or low) in both studies. It should be noted that one feature selection method and one classification method was removed from the analysis for the HNSCC study, and therefore they were also not considered while deciding thresholds for NSCLC study. All values of AUC and stability along with the group information are reported in Table 2 (feature selection methods) and Table 3 (classification methods).

**Table 2:** **Table describing the representative AUC and stability of feature selection methods**.

Feature selection method	AUC (HNSCC)	AUC (NSCLC)	Stability (HNSCC)	Stability (NSCLC)
RELF	0.62 ± 0.09 (High)	0.61 ± 0.04 (High)	0.63 ± 0.12 (Low)	0.91 ± 0.05 (High)
FSCR	0.63 ± 0.08 (High)	0.62 ± 0.04 (High)	0.51 ± 0.13 (Low)	0.78 ± 0.08 (High)
GINI	0.58 ± 0.07 (Low)	0.62 ± 0.04 (High)	0.66 ± 0.11 (High)	0.68 ± 0.10 (Low)
JMI	0.59 ± 0.07 (Low)	0.61 ± 0.04 (High)	0.67 ± 0.05 (High)	0.68 ± 0.05 (Low)
CIFE	0.68 ± 0.08 (High)	0.60 ± 0.03 (Low)	0.7 ± 0.04 (High)	0.69 ± 0.05 (Low)
DISR	0.56 ± 0.06 (Low)	0.62 ± 0.05 (High)	0.65 ± 0.08 (Low)	0.69 ± 0.05 (Low)
MIM	0.61 ± 0.08 (High)	0.61 ± 0.04 (High)	0.64 ± 0.1 (Low)	0.94 ± 0.02 (High)
CMIM	0.6 ± 0.07 (Low)	0.62 ± 0.04 (High)	0.71 ± 0.04 (High)	0.73 ± 0.04 (Low)
ICAP	0.59 ± 0.07 (Low)	0.61 ± 0.03 (High)	0.71 ± 0.03 (High)	0.72 ± 0.04 (Low)
TSCR	0.62 ± 0.07 (High)	0.61 ± 0.02 (High)	0.54 ± 0.01 (Low)	0.78 ± 0.12 (High)
MRMR	0.69 ± 0.07 (High)	0.63 ± 0.06 (High)	0.66 ± 0.03 (High)	0.74 ± 0.03 (High)
MIFS	0.66 ± 0.07 (High)	0.63 ± 0.06 (High)	0.69 ± 0.04 (High)	0.8 ± 0.03 (High)
WLCX	0.55 ± 0.06 (Low)	0.65 ± 0.02 (High)	0.71 ± 0.06 (High)	0.84 ± 0.05 (High)

*HNSCC thresholds: AUC = 0.61, stability = 0.66; NSCLC thresholds: AUC = 0.61, stability = 0.74*.

**Table 3:** **Table describing the representative AUC and stability of classification methods**.

Classification method	AUC (HNSCC)	AUC (NSCLC)	RSD% (HNSCC)	RSD% (NSCLC)
Nnet	0.59 ± 0.07 (Low)	0.57 ± 0.04 (Low)	11.54 (Low)	6.41 (Low)
DT	0.56 ± 0.05 (Low)	0.54 ± 0.04 (Low)	11.4 (High)	7.89 (Low)
BST	0.56 ± 0.07 (Low)	0.58 ± 0.04 (Low)	11.28 (High)	8.23 (Low)
BY	0.67 ± 0.06 (High)	0.64 ± 0.05 (High)	11.28 (High)	0.86 (High)
BAG	0.55 ± 0.06 (Low)	0.64 ± 0.03 (High)	9.27 (High)	5.56 (High)
RF	0.61 ± 0.06 (High)	0.66 ± 0.03 (High)	7.36 (High)	3.52 (High)
MARS	0.58 ± 0.05 (Low)	0.61 ± 0.03 (High)	12.47 (Low)	6.98 (Low)
SVM	0.64 ± 0.09 (High)	0.61 ± 0.03 (High)	12.69 (Low)	6.39 (Low)
NN	0.62 ± 0.05 (High)	0.61 ± 0.02 (High)	10.52 (High)	4.08 (High)
GLM	0.72 ± 0.08 (High)	0.63 ± 0.02 (High)	11.78 (Low)	2.19 (High)
PLSR	0.73 ± 0.07 (High)	0.63 ± 0.02 (High)	12.75 (Low)	2.24 (High)

*HNSCC thresholds: AUC = 0.61, RSD% = 11.4; NSCLC thresholds: AUC = 0.61, RSD% = 5.56*.

All analysis was done using R software (R Core Team, Vienna, Austria) version 3.1.2 and Matlab R2012b (The Mathworks, Natick, MA, USA) with Windows 7.

## Results

**Figure 1:**
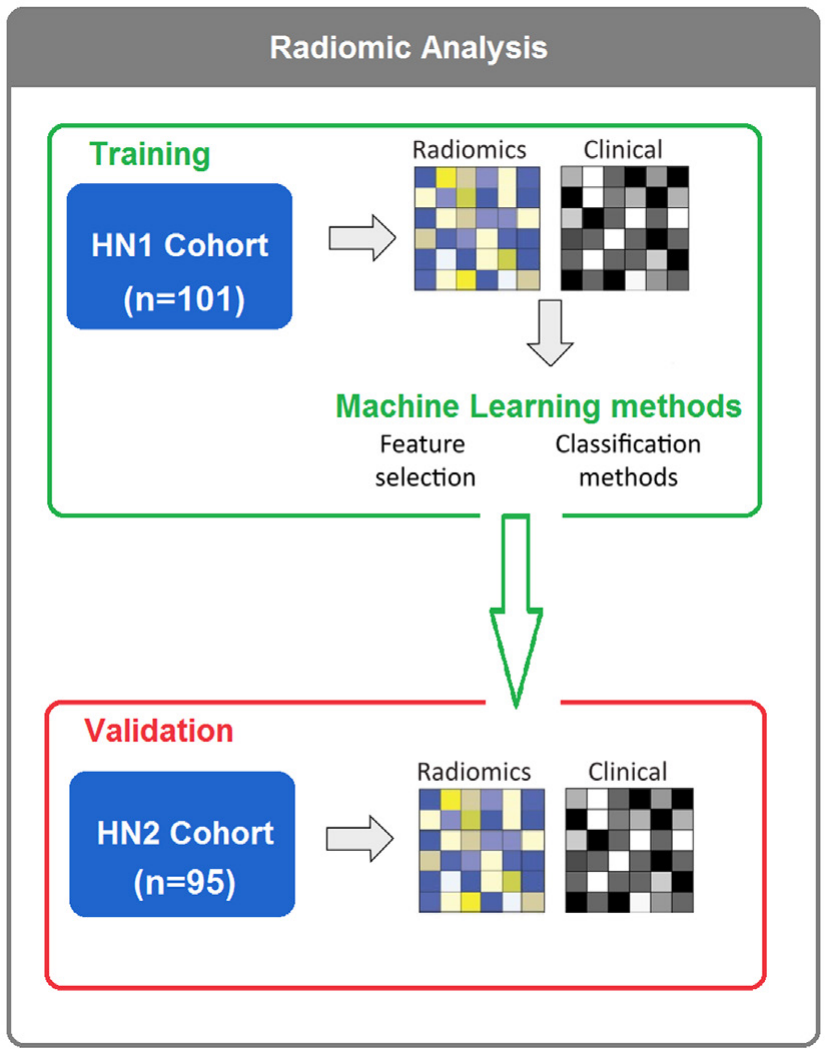
**In total, 196 HNSCC patients were considered**. Four hundred forty radiomic features were extracted from the segmented tumor regions of the CT images. Feature selection and classification training were done using the training cohort HN1 (*n* = 101), whereas HN2 (*n* = 95) cohort was used as a validation cohort.

To assess different machine-learning methods for radiomic survival models of head and neck cancer patients, we extracted 440 radiomic features from the segmented tumor regions of two independent HNSCC cohorts. Cohort HN1 (*n* = 101 patients) was used for feature selection and classification training, whereas the prediction performance was assessed using the validation cohort HN2 (*n* = 95 patients) (see Figure 1).

### Prognostic Performance

**Figure 2:**
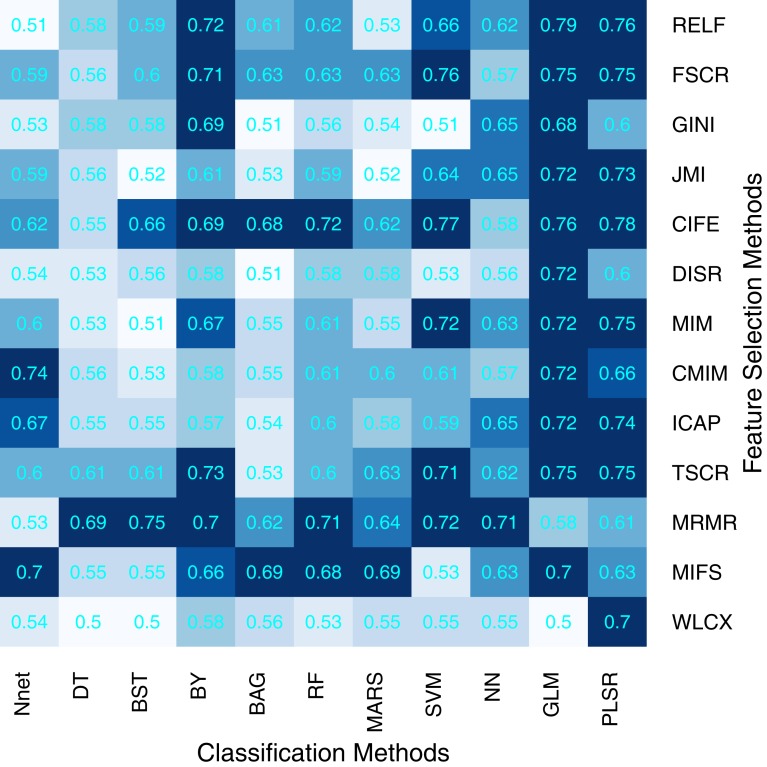
**Heatmap depicting the prognostic performance (AUC) of feature selection (in rows) and classification (in columns) methods**. It can be observed that PLSR and GLM classification methods and feature selection methods MRMR and MIFS shows relatively high prognostic performance in many cases.

We used area under receiver operating characteristics curve (AUC) to quantify the prognostic performance of different feature selection and classification methods. Figure 2 depicts the performance of feature selection (in rows) and classification methods (in columns) using the 30 top ranked features after applying feature selection. A median AUC of all 13 feature selection AUC values was used as the representative AUC of a classifier. Similarly, for each feature selection method, a median of 11 classification AUCs was used as the representative AUC. These representative AUC values for the feature selection and classification methods are given in Tables 2 and 3, respectively.

Feature selection methods, MRMR (AUC: 0.69 ± 0.07) and MIFS (AUC: 0.66 ± 0.07) showed high prognostic performance, whereas methods WLCX (AUC: 0.55 ± 0.06) and DISR (AUC: 0.56 ± 0.06) had lowest median AUCs.

In the case of classification methods, GLM (AUC: 0.72 ± 0.08) (median ± SD) and PLSR (AUC: 0.73 ± 0.07) had highest prognostic performance, whereas BAG (AUC: 0.55 ± 0.06), DT (AUC: 0.56 ± 0.05), and BST (AUC: 0.56 ± 0.07) showed lower AUC values. We repeated the above experiment by varying the number of selected features (range 5–50). Results with respect to 10, 20, 40, and 50 representative (top ranked) features are reported in supplement Figures S1–S4 in Supplementary Material. In addition, median AUC values over each of the experimental factors (feature selection methods, classification methods, and number of selected features) are depicted by the heatmaps in supplement Figures S5–S7 in Supplementary Material. Here as well, GLM and PLSR (classifiers) and MRMR and MIFS (feature selection methods) showed highest median AUCs in majority of cases.

### Stability

To assess the stability of feature selection methods against data perturbation, we used the hard data perturbation setting (37). We observed that WLCX (stability = 0.71 ± 0.06) (median ± SD), ICAP (stability = 0.71 ± 0.03), and CMIM (stability = 0.71 ± 0.04) showed high stability against data perturbation, whereas FSCR (stability = 0.51 ± 0.13) and TSCR (stability = 0.54 ± 0.10) had lower stability (Table 2).

In order to assess the stability of a classifier, we used the relative standard deviation (RSD) and a bootstrap approach. We observed that RF (RSD = 7.36%) and BAG (9.27%) were relatively more stable classification methods. PLSR (RSD = 12.75%) and SVM (RSD = 12.69%) showed higher RSD, which indicated lower stability of these methods. RSD (%) values corresponding to all 11 classifiers are reported in Table 3.

### Prognostic Performance and Stability

**Figure 3:**
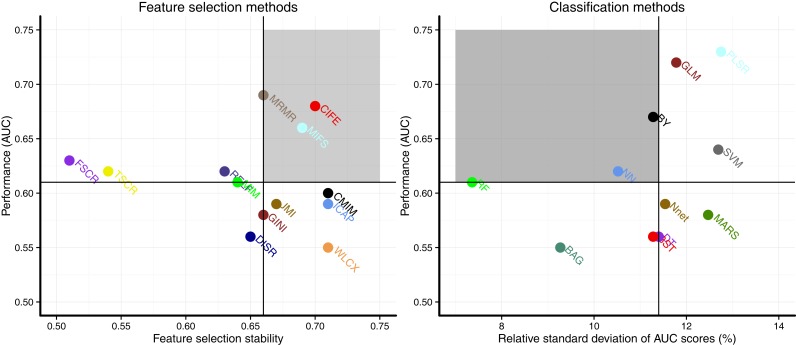
**Scatterplots of stability and prognostic performance (AUC) for feature selection (Left) and classification methods (right)**. Feature selection methods having stability ≥0.66 (median stability) and AUC ≥ 0.61 (median AUC) are considered as highly reliable and prognostic methods. Similarly, classification methods having RSD ≤ 11.4 (median RSD) and AUC ≥ 0.61 (median AUC) are considered as highly reliable and accurate ones. Highly reliable and prognostic methods are displayed in a gray square region.

Scatterplots in Figure 3 display the stability and prognostic performance of different feature selection and classification methods. It can be observed that the feature selection methods MRMR (AUC = 0.69 ± 0.07, stability = 0.66 ± 0.03), MIFS (AUC = 0.66 ± 0.07, stability = 0.69 ± 0.04), and CIFE (AUC = 0.68 ± 0.08, stability = 0.7 ± 0.04) showed higher prognostic performance and stability than the corresponding median values across all feature selection methods (AUC = 0.61, stability = 0.66). Similarly for classification methods, the stability and prognostic performance of RF (AUC = 0.61 ± 0.06, RSD = 7.36%), NN (AUC = 0.62 ± 0.05, RSD = 10.52%), and BY (AUC = 0.67 ± 0.06, RSD = 11.28%) were better than the corresponding median values (RSD = 11.4%, AUC = 0.61).

### Experimental Factors Affecting Radiomics-Based Survival Prediction

**Figure 4:**
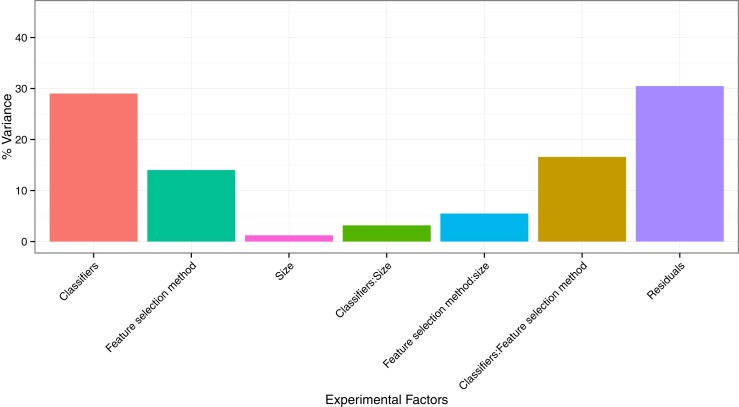
**Variation of AUC explained by the experimental factors and their interactions**. It can be observed that classification method was the most dominant source of variation in prediction score. Size of the selected (representative) feature subset shared the least of the total variance.

To quantify the effects of the three experimental factors (feature selection methods, classification methods, and the number of selected features), we performed multifactor ANOVA on AUC scores. We observed that all three experimental parameters are the significant factors affecting the prognostic performance (Figure 4). Classification method was the most dominant source of variability as it explained 29.02% of the total variance in AUC scores. Feature selection methods accounted for the 14.02%, whereas interaction of classifier and feature selection explained 16.59% of the total variance. Size of the selected (representative) feature subset only shared 1.22% of the total variance (Figure 4).

### Comparison with the NSCLC Cohorts

We compared the obtained results related to feature selection and classification methods to the previously published study of NSCLC cohorts (30) (see Tables 2 and 3).

Two feature selection methods MRMR and MIFS displayed high prognostic performance and stability across both the cancer types. Methods RELF and MIM showed high prognostic power in both the cancer types. However, they had marginally low stability for HNSCC cohorts. Interestingly, WLCX had highest prognostic performance for NSCLC cohorts, whereas it showed lowest prognostic performance for HNSCC cohorts. However, in both the cancer types WLCX displayed high stability against data perturbation (see Table 2).

Three classification methods BY, RF, and NN showed high prognostic performance and stability across both cancer types. PLSR and GLM had high prognostic performance in both cancer types, but these two methods showed low stability for HNSCC cohorts. Classifier BAG displayed lowest prognostic power in HNSCC radiomic cohorts, whereas it had the second highest performance in NSCLC cohorts. It should be noted that the stability of BAG was high in both cancer types (see Table 3).

## Discussion

The applications of medical imaging in cancer diagnostics and treatment planning have expanded greatly over the time. Moreover, developments in imaging technologies and computational approaches have led the emergence of “radiomics,” which is a high-throughput medical image data mining field (1). Radiomics is a non-invasive and cost-effective medical informatics approach, which provides unprecedented opportunities for improvising clinical decision support (2). Hence, in the context of radiomics, medical imaging is expected to have a more central role in cancer care (1, 2, 26). With increasing cohort sizes and expanding feature dimensions, radiomics targets a large pool of medical imaging data (“Big data”). Automated, reliable, and efficient methods are desired to extract and mine the most relevant information from these large radiomic cohorts. A recently published study (30) has articulated the scope and applicability of different machine-learning methods in two independent radiomic cohorts of patients with NSCLC. They proposed an unbiased framework to compare the prognostic performance and stability of different machine-learning methods. It was recommended that these different machine-learning methods should be further evaluated in different cancer types and with respect to different radiomic cohorts (30). Furthermore, it has been previously shown that the grouping and prognostic characteristics of radiomic features are cancer specific (16). Therefore, the primary objective of our study was to assess the state of the art machine-learning methods in two independent HNSCC cohorts.

Our analysis quantifies the prognostic power and stability of different machine-learning methods for the survival prediction of head and neck cancer patients. Depending on the requirement, one may prefer higher prognostic power or stability and choose the methods accordingly. Considering the stability and prognostic performance together, three feature selection methods MRMR, MIFS, and CIFE and three classification methods BY, RF, and NN should be preferred for head and neck radiomic analyses as they displayed relatively higher prognostic power and stability than other methods.

Assessing the variability in prediction performance by multifactor ANOVA, we found that the classification method is the most dominant source of variation in prediction performance, and hence it should be chosen carefully. Size of the selected feature subset contributed the least in the AUC variation.

Comparing the results of this study with the previously published study of NSCLC, we observed that except BAG and MARS, all classifiers showed consistency in their prognostic performance across the two cancer types. However, it should be noted that the AUC values for MARS were quite close to the threshold (median AUC). In the case of feature selection methods, WLCX showed no prediction consistency across the two cancer types. Besides that, methods CIFE, DISR, GINI, CMIM, and ICAP also showed no consistency in the grouping based on prognostic performance. However, it should be noted that for methods CMIM and ICAP, the AUC values did not differ much from the threshold (median AUC). As far as stability is concerned, except for classifiers PLSR, GLM, DT, and BST, all classifiers showed consistency in their stability-based grouping. For feature selection methods, only MRMR, MIFS, WLCX, and DISR showed consistent stability-based grouping across both the studies. It should be noted that for HNSCC cohorts, stability values for all the methods (classification and feature selection) were lower than the ones obtained in NSCLC cohorts. The intuitive explanation for this could be the smaller cohort size. The training cohort in NSCLC study (30) had almost three times more samples than the training cohort of our HNSCC study.

Considering the stability and prognostic performance together and comparing the results between the two cancer types (HNSCC and NSCLC), we observed that the three classifiers BY, RF, and NN had high stability and high prognostic performance in both HNSCC and NSCLC studies. Similarly, two feature selection methods MRMR and MIFS showed consistently high values of AUC and stability in both cancer types. These results indicate that such methods should be first preferences for radiomics-based prognostic analyses due to their consistency. A note of caution: different methods are categorized into high/low (prediction performance and stability) group based on simple thresholds (median AUC and median stability). These thresholds are no gold standard and they are only used for comparing the performances of different machine-learning methods in a relative manner. It can be observed from the results [Figure 3; Figure 3 in Ref. (30)] that some of the so-called “not consistent” methods are quite close to the thresholds, and they should not be neglected completely. Further validation of these methods with different clinical outcomes, different imaging modalities, and also different radiomic cohorts could provide better insights about their applicability.

Results related to the variability of AUC scores were comparable in both the cancer types as in both studies, classification method contributed highest and size contributed the least in the performance variance. Interestingly, for HNSCC cohorts, feature selection method contributed almost two times more in the AUC variation than in the case of NSCLC study (30).

As mentioned previously (30), the machine-learning methods used in this analysis were chosen because of their simplicity, efficiency, and popularity in the literature. Furthermore, an interesting discussion about the publicly available implementation tools and the used parameter configurations were presented before (30).

It has been shown that statistical models based on patient’s tumor and treatment characteristics provide significantly better predictions/prognosis than human expert (38). Therefore, radiomics-based machine-learning models could be vital for clinical decision support. However, there are few inherent challenges with radiomics. Several studies have investigated the variability of radiomic features with respect to different imaging scanners (39, 40), tumor delineation methods (13, 41), reconstruction methods (42), discretization (43), etc. These different sources of variability need to be considered for radiomics-based analyses. For example, normalizing or standardizing the features could help in reducing batch effects. Furthermore, the performance of prediction models should be tested in independent validation cohorts. As far as our study is concerned, we standardized all features using *Z*-score standardization. Moreover, we used an independent validation cohort to assess the prediction performance of different machine-learning methods.

The potential clinical utility of radiomics-based prognostic models has been highlighted before (2). With the expanding radiomic cohorts and feature dimensions, as well as by integrating different biological and clinical information together with radiomics, higher prognostic performance could be achieved. In this regard, our studies could be an important reference as we compared a large panel of machine-learning methods across two different cancer types. The prognostic power and stability of different machine-learning methods were compared using four independent radiomic cohorts. Such a comparative investigation could help in identifying the optimal and reliable machine-learning methods for radiomics-based prognostic analyses, which overall could broaden the scope of radiomics in cancer care.

## Author Contributions

HA, CP, and PG conceived of the project, analyzed the data, and wrote the paper. DR, MR, and PL provided expert guidance, data, or analysis tools and reviewed the manuscript.

## Conflict of Interest Statement

The authors declare that the research was conducted in the absence of any commercial or financial relationships that could be construed as a potential conflict of interest.
