# Supplementary material for: Radiomic Machine-Learning Classifiers for Prognostic Biomarkers of Head and Neck Cancer
Source: Front Oncol. 2015 Dec 3;5:272. doi: 10.3389/fonc.2015.00272 (PMC4668290; doi:10.3389/fonc.2015.00272)
Supplement: Supplementary file 1 [file Data_Sheet_1.DOCX]

Supplementary Material

Radiomic Machine Learning Classifiers for Prognostic Biomarkers of Head & Neck Cancer

**Chintan Parmar^1,2,3,*,#^, Patrick Grossmann^1,2,4,#^, Derek Rietveld^5^, Michelle M. Rietbergen^6^, Philippe Lambin^3^, Hugo J.W.L. Aerts^1,2,4,*^**

^1^Departments of Radiation Oncology and ^2^Radiology, Dana-Farber Cancer Institute, Brigham and Women’s Hospital, Harvard Medical School, Boston, MA, USA.

^3^Radiation Oncology (MAASTRO), Research Institute GROW, Maastricht University, Maastricht, The Netherlands

^4^Department of Biostatistics & Computational Biology, Dana-Farber Cancer Institute, Boston, MA, USA

^5^Department of Radiation Oncology, VU University Medical Center, Amsterdam, The Netherlands

^6^Department of Otolaryngology/Head and Neck Surgery, VU University Medical Center, Amsterdam, The Netherlands

**# Equal contribution**

*** Correspondence:** Hugo Aerts or Chintan Parmar, Dana-Farber Cancer Institute, Brigham and Women’s Hospital, Harvard Medical School, 450 Brookline Ave, JF518, Boston, MA, 02115-5450, USA

[Hugo_Aerts@dfci.harvard.edu](mailto:Hugo_Aerts@dfci.harvard.edu), [Chintan_Parmar@dfci.harvard.edu](mailto:Chintan_Parmar@dfci.harvard.edu)

# Supplementary Figures

**Figure S1.**  Prognostic performance (AUC) of feature selection (in rows) and classification methods (in columns) with top 10 selected features.

**

**Figure S2.** Prognostic performance (AUC) of feature selection and classification methods with top 20 selected features.

**

**Figure S3.** Prognostic performance (AUC) of feature selection and classification methods with top 40 selected features.

**Figure S4 |** Prognostic performance (AUC) of feature selection and classification methods with top 50 selected features.

**Figure S5.** Prognostic performance (median over all feature selection methods) corresponding to classification methods (in columns) and the number of selected features (in rows).

**Figure S6.** Prognostic performance (median over all classification methods) corresponding to feature selection methods (in rows) and the number of selected features (in columns).

**Figure S7.** Prognostic performance (median over the number of selected features) corresponding to classification methods (in columns) and feature selection methods (in rows).
